# Hypermethylation of *CDKN2A* exon 2 in tumor, tumor-adjacent and tumor-distant tissues from breast cancer patients

**DOI:** 10.1186/s12885-017-3244-2

**Published:** 2017-04-12

**Authors:** Melanie Spitzwieser, Elisabeth Entfellner, Bettina Werner, Walter Pulverer, Georg Pfeiler, Stefan Hacker, Margit Cichna-Markl

**Affiliations:** 1 Department of Analytical Chemistry, University of Vienna, Währinger Str. 38, 1090 Vienna, Austria; 2 Molecular Diagnostics, Austrian Institute of Technology, Muthgasse 11, 1190 Vienna, Austria; 3 Department of Obstetrics and Gynecology, Division of Gynecology and Gynecological Oncology, Medical University of Vienna, Währinger Gürtel 18-20, 1090 Vienna, Austria; 4 Department of Plastic and Reconstructive Surgery, Medical University of Vienna, Währinger Gürtel 18-20, 1090 Vienna, Austria

**Keywords:** DNA methylation, *CDKN2A*, Exon 2, Tumor suppressor genes, Breast cancer, Field cancerization, Tumor-adjacent tissue, Tumor-distant tissue, Methylation-sensitive high resolution melting, Pyrosequencing

## Abstract

**Background:**

Breast carcinogenesis is a multistep process involving genetic and epigenetic changes. Tumor tissues are frequently characterized by gene-specific hypermethylation and global DNA hypomethylation. Aberrant DNA methylation levels have, however, not only been found in tumors, but also in tumor-surrounding tissue appearing histologically normal. This phenomenon is called field cancerization. Knowledge of the existence of a cancer field and its spread are of clinical relevance. If the tissue showing pre-neoplastic lesions is not removed by surgery, it may develop into invasive carcinoma.

**Methods:**

We investigated the prevalence of gene-specific and global DNA methylation changes in tumor-adjacent and tumor-distant tissues in comparison to tumor tissues from the same breast cancer patients (*n* = 18) and normal breast tissues from healthy women (*n* = 4). Methylation-sensitive high resolution melting (MS-HRM) analysis was applied to determine methylation levels in the promoters of *APC*, *BRCA1*, *CDKN2A (p16)*, *ESR1*, *HER2/neu* and *PTEN,* in *CDKN2A* exon 2 and in LINE-1, as indicator for the global DNA methylation extent. The methylation status of the *ESR2* promoter was determined by pyrosequencing.

**Results:**

Tumor-adjacent and tumor-distant tissues frequently showed pre-neoplastic gene-specific and global DNA methylation changes. The *APC* promoter (*p* = 0.003) and exon 2 of *CDKN2A* (*p* < 0.001) were significantly higher methylated in tumors than in normal breast tissues from healthy women. For both regions, significant differences were also found between tumor and tumor-adjacent tissues (*p* = 0.001 and *p* < 0.001, respectively) and tumor and tumor-distant tissues (*p* = 0.001 and *p* < 0.001, respectively) from breast cancer patients. In addition, tumor-adjacent (*p* = 0.002) and tumor-distant tissues (*p* = 0.005) showed significantly higher methylation levels of *CDKN2A* exon 2 than normal breast tissues serving as control. Significant correlations were found between the proliferative activity and the methylation status of *CDKN2A* exon 2 in tumor (*r* = −0.485, *p* = 0.041) and tumor-distant tissues (*r* = −0.498, *p* = 0.036).

**Conclusions:**

From our results we can conclude that methylation changes in *CDKN2A* exon 2 are associated with breast carcinogenesis. Further investigations are, however, necessary to confirm that hypermethylation of *CDKN2A* exon 2 is associated with tumor proliferative activity.

## Background

Breast cancer is the second most common cancer in the total world population and the most frequent cancer among women. In more developed countries, it is the second cause and in less developed regions, even the most frequent cause of cancer death in women [1]. Outcome and survival of breast cancer patients critically depend on the time of diagnosis of the malignant lesion.

Breast carcinogenesis is a multistep process involving not only genetic but also epigenetic changes, in particular aberrations in the extent of DNA methylation [2–4]. Tumors of many cancer types including breast cancer are frequently characterized by the co-occurrence of global DNA hypomethylation and gene-specific hypermethylation [5]. Hypermethylation of CpG dinucleotides (CpGs) in the promoter region of genes frequently results in transcriptional silencing [6] whereas global DNA hypomethylation is linked to chromosomal instability [7]. Several studies have already evaluated the applicability of gene-specific hypermethylation as diagnostic [8, 9], prognostic [10–12] or predictive [13, 14] biomarker in breast cancer.

Tissues surrounding tumors frequently appear histologically normal but show pre-neoplastic lesions. This phenomenon is called “field cancerization” or “field effect” [15]. Aberrant DNA methylation levels in tumor-adjacent tissues have been reported for various cancer types including colorectal [16], prostate [17, 18] and breast cancer [19–23]. Knowledge of the existence of a cancer field is of clinical relevance. If the tissue showing pre-neoplastic lesions is not removed by surgery, it may develop into invasive carcinoma.

In a previous study, we determined the promoter methylation status of six tumor suppressor genes (*CCND2*, *DAPK1*, *GSTP1*, *HIN-1*, *MGMT* and *RASSF1A*) in tumor, tumor-adjacent and tumor-distant tissues from breast cancer patients and normal breast tissues from healthy controls [24]. Promoter methylation levels of *HIN-1*, *MGMT* and *RASSF1A* were found to be potential biomarkers for detecting field cancerization in breast cancer patients.

In the present study, we also investigated the prevalence of pre-neoplastic DNA methylation changes in breast cancer patients. The set of breast tissue samples used previously [24] was analyzed for the methylation status of the following seven genes: adenomatous polyposis coli (*APC*); breast cancer 1, early onset (*BRCA1*); cyclin-dependent kinase inhibitor 2A (*CDKN2A, p16*)*;* estrogen receptor α (*ESR1*); estrogen receptor β (*ESR2)*; human epidermal growth factor receptor 2 (*HER2/neu*); and phosphatase and tensin homolog (*PTEN*). These genes were selected because they have previously been associated with breast cancer [25–29]. We focused on determining the methylation status in the promoter region of these genes; in case of *CDKN2A,* we were, however, also interested in exon 2. Hypermethylation of *CDKN2A* exon 2 has previously been linked to late stage oesophageal cancer [30]. To the best of our knowledge, methylation levels for *CDKN2A* exon 2 in tissues from breast cancer patients have not been published so far. In addition to the gene-specific methylation status, we assessed the global DNA methylation status by using LINE-1 (long interspersed element 1; retrotransposable element 1) as indicator. Statistical analyses were carried out to test if there are significant differences in the gene-specific and/or the global DNA methylation status between tumor, tumor-adjacent and tumor-distant tissues from breast cancer patients and normal breast tissues from healthy women. In addition, we checked if the methylation status of any of the investigated regions is linked to clinicopathological parameters such as histologic type, histological grading, B classification, proliferative activity (MIB-1) and molecular subtype of the tumor.

In order to get a broader picture of the prevalence of pre-neoplastic DNA methylation changes in tumor-surrounding tissues in breast cancer patients, data that we published previously for the same set of breast tissue samples [24, 31] was included in part of the statistical tests.

## Methods

### Breast tissue samples

Biopsy samples were collected from 18 breast cancer patients at diagnosis of the disease (age at diagnosis: 39-76 years, mean age at diagnosis: 58 years). None of the patients had a family history of breast cancer. By ultrasound guided needle biopsy, three tissue samples were taken from each patient: the first sample directly from the tumor, the second one about 1 cm from the tumor center (“tumor-adjacent tissue”) and the third one at least 3 cm from the tumor center (“tumor-distant tissue”). Non-cancerous breast tissue samples were taken from four women (aged from 44 to 60 years; mean age: 53 years) during breast reductive surgery. From two of these women, samples of left and right breast were available. In case of *CDKN2A* exon 2, we additionally analyzed breast tissue samples (left and right breast) from further three healthy women. The study was approved by the Ethics Commission of the Medical University of Vienna (application number 1074/2011). All participants gave written informed consent.

### Characteristics of breast cancer patients

Table 1 summarizes the characteristics of the breast cancer patients, including menopause status, histologic type, histological grading, B classification, proliferative activity (MIB-1), status of estrogen receptor (ER), progesterone receptor (PR) and human epidermal growth factor receptor 2 (HER2/neu) as well as the molecular subtype.

**Table 1:** Clinical and pathological characteristics of breast cancer patients

Patient	Menopause status	Histologic type	Histological grading	B classification	MIB-1 [%]	Receptor status			Molecular subtype
						ER	PR	HER2/neu	
1	Post	IDC	G2	B5b	10	+++	++	−	Luminal A
2	Post	IDC	G2	B5b	10	+++	++	−	Luminal A
3	Peri	IDC	G3	B5b	30	+++	−	−	Luminal A
4	Pre	IDC	G2	B5b	40	+++	++	+++	Luminal B
5	Post	IDC	G2	B5b	60	+++	++	−	Luminal A
6	Pre	IDC	G3	B5b	50	+++	+++	+++	Luminal B
7	Post	IDC	G3	B5b	20	pos.	pos.	−	Luminal A
8	Post	IDC	G2	B5b	20	+++	+++	−	Luminal A
9	Post	IDC	G3	B5	30	++	+++	−	Luminal A
10	Post	IDC	G3	B5b	20	+++	+++	+	Luminal B
11	Post	IDC	G1	B5c	20	+++	+++	+	Luminal B
12	Post	IDC	G3	B5b	70	−	−	−	Triple negative
13	Pre	ILC	n.s.	B5b	50	+++	++	−	Luminal A
14	Pre	IDC	G3	B5b	80	++	−	−	Luminal A
15	Post	IDC	G3	B5b	40	+++	++	−	Luminal A
16	Post	ILC	G2	B5b	30	+++	+++	−	Luminal A
17	Pre	Mucinous	G2	B5b	50	+++	++	+++	Luminal B
18	Post	IDC	G3	B5b	50	−	−	+++	HER2/neu

*IDC* invasive ductal carcinoma, *ILC* invasive lobular carcinoma, *MIB-1* mindbomb E3 ubiquitin protein ligase 1 (proliferative activity), *ER* estrogen receptor, *PR* progesterone receptor, *HER2/neu* human epidermal growth factor receptor 2, *n.s.* not specified, + weakly positive, ++ moderately positive, +++ strongly positive, − negative

### Breast cancer cell lines

Breast cancer cell lines MCF-7, MDA-MB-231 and ZR-75-1 were grown in Dulbecco’s Minimal Essential Medium (DMEM), Leibovitz’s L-15 and RPMI-1640, respectively. Culture media were supplemented with 10% fetal calf serum (PAA, Austria). Cell cultures were periodically checked for mycoplasma contamination.

### DNA extraction

Genomic DNA was extracted using the QIAamp DNA Mini Kit (Qiagen, Germany) according to the manufacturer’s recommendations. The DNA concentration was determined with a Nanodrop 2000c spectrophotometer (Thermo Scientific, USA).

### Bisulfite conversion

DNA extracted from biopsy samples and commercially available human control DNA (CpGenome Universal Methylated DNA, Millipore, USA and EpiTect Control DNA (human), unmethylated, Qiagen) were treated with sodium bisulfite using the EpiTect Fast Bisulfite Kit (Qiagen) following the manufacturer’s protocol.

### Methylation-sensitive high resolution melting (MS-HRM)

Primer sequences for *APC* [32], *BRCA1* [33], *CDKN2A* (promoter) [34] and *PTEN* [35] were taken from literature, those for *CDKN2A* (exon 2), *ESR1, HER2/neu* and LINE-1 were designed in-house. Nucleotide sequences were obtained from the National Center for Biotechnology Information (NCBI; [36]) database. The promoter region of the genes was identified using the Transcriptional Regulatory Element Database (TRED; [37]) or the Eukaryotic Promoter Database (EPD; [38]). Primers were designed with the Methyl Primer Express Software v1.0 (Applied Biosystems, USA). For each MS-HRM method, the annealing temperature (T_a_) and the additional MgCl_2_ concentration were optimized in-house. Sequences of the MS-HRM primers and their optimized conditions are summarized in Table 2.

**Table 2:** Primer sequences and conditions for MS-HRM analysis

	Primer sequence	Primer concentration [nM]	Additional MgCl_2_ concentration [mM]	T_a_ [°C]^a^	Amplicon length [bp]	No. of CpGs^b^	LOD/LOQ [%]	Reference
*APC*	f: 5′ AAGTAGTTGTGTAATTCGTTGGAT 3′ r: 5′ CACCTCCATTCTATCTCCAATA 3′	500	-	53	149	10	0.4/1.4	[32]
*BRCA1*	f: 5′ TTGTTGTTTAGCGGTAGTTTTTTGGTT 3′ r: 5′ AACCTATCCCCCGTCCAAAAA 3′	250	2	61	122	9	0.4/1.6	[33]
*CDKN2A*	f: 5′ CGGAGGAAGAAAGAGGAGGGGT 3′ r: 5′ CGCTACCTACTCTCCCCCTCT 3′	400	-	62	93	7	1.0/3.3	[34]
*CDKN2A* (exon 2)	f: 5′ GGCGGAGTTGTTGTTGTTTTATG 3′ r: 5′ ACAACACCACCAACGTATCCAA 3′	250	1.5	52	116	10	3.5/10.7	In-house [NG_007485.1]
*ESR1*	f: 5′ TTAAAGTTGGAGGTTCGGGAGT 3′ r: 5′ CTACCCCGAAAACCTACGAATC 3′	250	-	52	112	8	0.1/0.5	In-house [NG_008493]
*HER2/neu*	f: 5′ GGATTGGAGAAATTAGGGGAGT 3′ r: 5′ ACTCCGACTAAACCCGACTAAA 3′	250	2	52	137	15	0.4/1.4	In-house [NG_007503.1]
LINE-1	f: 5′ TGTTAGATAGTGGGTGTAGGTT 3′ r: 5′ AAATACATCCGTCACCCCTTT 3′	250	-	57	139	9	11.7/22.3	In-house [X58075.1]
*PTEN*	f: 5′ TCGGTTGGGTTTTTGGGTAGAGG 3′ r: 5′ CGCAAACTCTACTAAACATACCCAATAT 3′	250	-	60	156	9	0.1/0.5	[35]

*f* forward primer, *r* reverse primer, *T*_*a*_ annealing temperature, *bp* base pairs, *LOD* limit of detection, *LOQ* limit of quantification

^a^Touchdown in 1 °C increments in cycles 1-7

^b^Without CpGs in the primers

Polymerase chain reaction (PCR) and MS-HRM analysis were carried out using a Rotor-Gene Q instrument (Qiagen) and the EpiTect HRM PCR Kit (Qiagen). The reaction mixture per well had a total volume of 20 μl and included 10 μl 2× EpiTect HRM PCR Master Mix (Qiagen), varying amounts of MgCl_2_, forward and reverse primer, RNase-free water and 10 ng of bisulfite converted DNA. Amplification was performed under the following conditions: initial PCR activation step at 95 °C for 5 min followed by 50 cycles at 95 °C for 10 s, T_a_ of the respective primer set for 30 s (Table 2) and 72 °C for 10 s; denaturation step at 95 °C for 1 min followed by a hybridization step at 40 °C for 1 min. PCR was directly followed by a HRM step where the temperature was increased by 0.1 °C increments per 2 s.

MS-HRM data was evaluated with the Rotor-Gene Q Series Software 2.1.0 (Qiagen). The DNA methylation status was determined with the help of calibration curves established by analyzing DNA standards differing in their methylation status. These standards were prepared by mixing unmethylated and methylated human control DNA in different proportions. From the normalized HRM curves we calculated the average of the normalized fluorescence signal for each standard over the entire temperature. Calibration functions were established with SigmaPlot 11.0 (Systat Software Inc., USA). Limit of detection (LOD) and limit of quantification (LOQ) of the MS-HRM methods were determined by repeatedly analyzing unmethylated control DNA. We calculated the mean and the standard deviation. The LOD (signal-to-noise ratio 3) was determined by adding three times the standard deviation and the LOQ (signal-to-noise ratio 10) by adding ten times the standard deviation to the mean.

### Pyrosequencing (PSQ)

Primer sequences for *ESR2* were designed in-house using the PyroMark Assay Design Software 2.0.1.15 (Qiagen). The nucleotide sequence was taken from the NCBI database, the promoter region was identified using TRED. Primer sequences are listed in Table 3.

**Table 3:** Primer sequences and conditions for pyrosequencing analysis

Gene	Primer sequence	Primer concentration [nM]	T_a_ [°C]	Amplicon length [bp]	No. of CpGs analyzed	Chromosome, position of sequence to analyze, DNA strand	Reference
*ESR2*	f: 5′ GGAGGTTGAGAGAAATAATTGTTTTTTGA 3′	400	61	255	9	14, [49,292- 49,369], positive strand	in-house [NG_011535.1]
	r: 5′ [Btn]ATAAACACACCCACCTTACCTTCTCTA 3′ s: 5′ GAAATAATTGTTTTTTGAAATTTG 3′						

*f* forward primer, *r* reverse primer, *s* sequencing primer, [*Btn*] biotin, *T*_*a*_ annealing temperature

PCR was performed in the Rotor-Gene Q instrument using the PyroMark PCR Kit (Qiagen). Each PCR reaction consisted of 12.5 μl PyroMark PCR Master Mix (2×), CoralLoad Concentrate (10×), forward and reverse primer, RNase-free water and 10 ng of bisulfite converted DNA. PCR conditions were as follows: initial activation step at 95 °C for 15 min, 50 cycles: 30 s at 94 °C, 30 s at 61 °C, 30 s at 72 °C and a final extension at 72 °C for 10 min. Pyrosequencing analyses were performed in the PyroMark Q24 Advanced instrument (Qiagen) by using PyroMark Q24 Advanced CpG Reagents (Qiagen). Sample preparation was carried out with the PyroMark Q24 Vacuum Workstation (Qiagen) as described previously [39].

### Agarose gel electrophoresis

To check identity and purity, PCR products randomly selected were loaded onto a 2% agarose gel in 1× TAE buffer, stained with GelRed (Biotium, USA) and visualized with an UVT-20 M transilluminator (Herolab, Germany).

### Statistical analysis

In MS-HRM analysis, bisulfite-treated DNA from breast tissue samples was analyzed in duplicates on at least two different days. In PSQ analysis, the bisulfite treated DNA was subjected to at least two PCR reactions. Each PCR product was subsequently sequenced once.

Methylation levels were treated either as categorical variables (MS-HRM methods: < LOD, < LOQ or ≥ LOQ; PSQ method: < LOQ or ≥ LOQ) or as continuous variables. If the methylation status was treated as continuous variable, a methylation status < LOD or < LOQ was substituted with a default value, namely half the LOD or half the LOQ, respectively, as proposed previously [40].

Statistical analyses were carried out with IBM SPSS Statistics Version 21.0. A *p* value < 0.05 (two-sided) was considered significant. Chi-squared test was used to evaluate if the methylation status is associated with any of the patients clinicopathological parameters. One-way ANOVA (analysis of variance), followed by post-hoc Tukey’s HSD (honest significant difference) test, was applied to test for significant differences in the DNA methylation status between tumor, tumor-adjacent and tumor-distant tissues as well as normal tissues from the healthy control group. Pearson’s correlation coefficient was used to assess the relationship between two continuous variables.

For principal component analysis (PCA), Qlucore’s Omics Explorer V3.2 (64bit) was used. The continuous methylation data was loaded into the software and each variable was normalized to mean 0 and variance 1. Ordering of the samples was based on the total variance captured by each component.

## Results

### MS-HRM and PSQ analysis

**Fig. 1:**
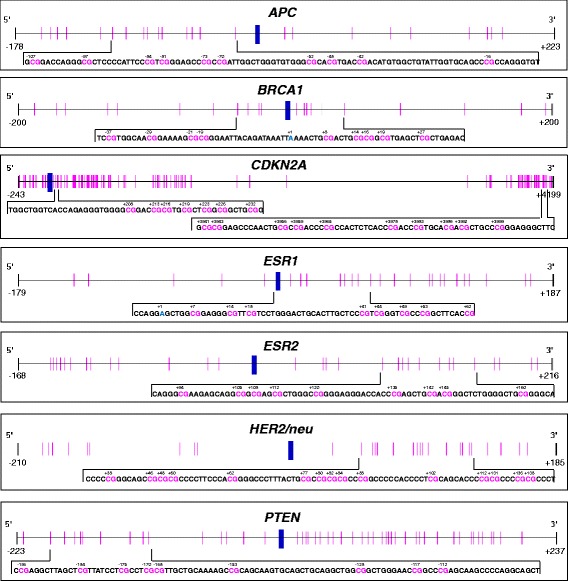
Schematic representation of the positions of the CpGs targeted by the MS-HRM and PSQ assays. The transcription start site (TSS, +1) is indicated by a *blue* vertical bar, the positions of the CpGs by *pink* vertical lines

MS-HRM assays for determining the methylation status in the promoter regions of *ESR1* and *HER2/neu,* exon 2 of *CDKN2A* and LINE-1 were developed and optimized in-house. The same holds for the PSQ method allowing the determination of the methylation status of the *ESR2* promoter. MS-HRM assays for the promoters of *APC, BRCA1, CDKN2A* and *PTEN* were taken from literature. Primer sequences and experimental conditions are summarized in Tables 2 and 3. Figure 1 indicates the position of the CpGs targeted by the assays in relation to the respective transcription start site (TSS).

**Fig. 2:**
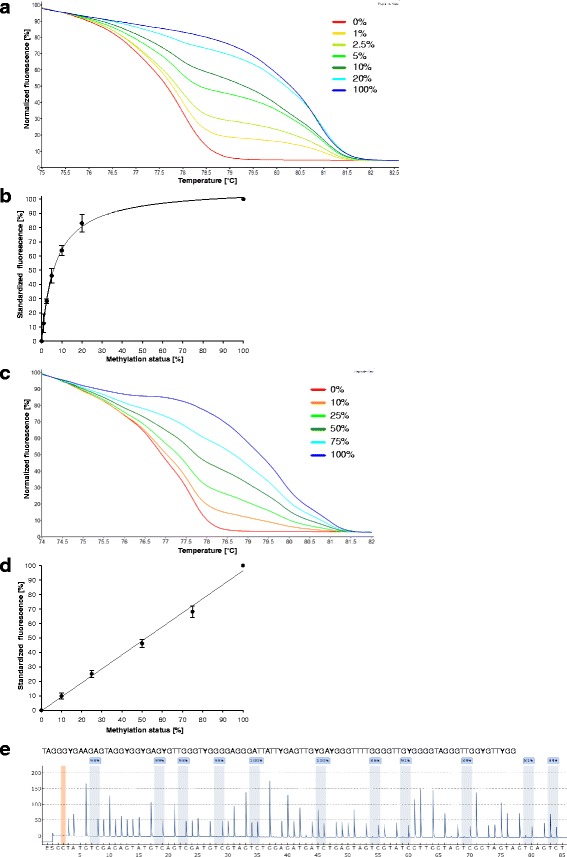
Analysis of DNA standards by MS-HRM (**a**–**d**) and PSQ (**e**). **a**, **c**: Normalized melting curves for *ESR1* (**a**) and *CDKN2A* (exon 2) (**c**) obtained by analyzing mixtures of unmethylated and fully methylated control DNA. Replicate view of duplicate measurements carried out on one day. **b**, **d**: Calibration curves for *ESR1* (**b**) and *CDKN2A* (exon 2) (**d**) obtained by repeatedly analyzing control DNA standards (*ESR1*: *n* = 6; *CDKN2A* (exon 2): *n* = 8). **e**: Representative pyrogram for *ESR2* obtained by analyzing methylated control DNA. Peaks highlighted in *blue* indicate the methylation status of the CpGs in the sequence to analyze. The position highlighted in *orange* serves as control for complete bisulfite conversion

Figure 2 shows representative normalized melting curves of DNA standards obtained with the MS-HRM assays for *ESR1* (a) and *CDKN2A* exon 2 (c). The corresponding calibration curves are shown in Fig. 2b and d, respectively. Each of the MS-HRM assays applied in the present study was validated with regard to limit of detection (LOD; S/*N* = 3), limit of quantification (LOQ; S/*N* = 10) and inter-day repeatability. LOD and LOQ were determined by repeatedly analyzing bisulfite-treated, unmethylated control DNA, the inter-day repeatability by analyzing mixtures of bisulfite-treated methylated and unmethylated control DNA on different days. With the exception of the assay for *CDKN2A* exon 2, the MS-HRM assays showed a PCR bias towards methylated alleles in PCR amplification, resulting in very low LODs and LOQs in the range from 0.1 to 1.0% and 0.5 to 3.3%, respectively (Table 2). LODs and LOQs of the MS-HRM assays for *CDKN2A* exon 2 and LINE-1 were 3.5% and 10.7% and 11.7% and 22.3%, respectively. The calibration curves (Fig. 2b and d) demonstrate the high inter-day repeatability of the assays. Figure 2e shows a representative pyrogram obtained with the PSQ assay for *ESR2*. By repeatedly amplifying unmethylated control DNA and subjecting the amplicons to pyrosequencing, the LOQ was determined to be 5% which is in line with the LOQ given by the manufacturer of the pyrosequencing instrument used.

### DNA methylation status in breast tissues from healthy controls

DNA extracts from breast tissues of four healthy women were used to determine base levels of DNA methylation in non-cancerous breast tissues. In all breast tissue samples, *BRCA1* and *HER2/neu* were unmethylated (methylation status < LOD), whereas *ESR2* showed methylation levels between LOD and LOQ. *PTEN* was slightly methylated (methylation status < LOQ) in three breast tissue samples. Methylation levels slightly ≥ LOQ were only obtained for *APC* in one and for *ESR1* in three samples. In case of *CDKN2A* exon 2, we analyzed breast tissue samples from seven healthy women. From five women, samples of left and right breast were available. In six healthy women, the methylation status of *CDKN2A* exon 2 was found to be < LOQ, in one woman it was even < LOD. The global methylation status was in the range from 88.4 to 90.7%. No differences were found between the tissue samples originating from the same women, neither in the gene-specific nor in the global methylation status.

### DNA methylation status in tumor, tumor-adjacent and tumor-distant tissues from breast cancer patients

**Fig. 3:**
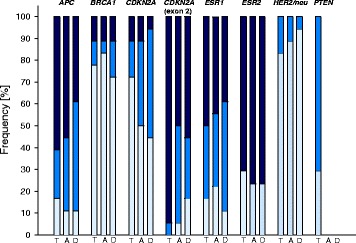
Frequency of gene-specific DNA methylation in tumor (T), tumor-adjacent (A) and tumor-distant (D) tissues. *Light blue*: methylation status < LOD, *middle blue*: methylation status < LOQ, *dark blue*: methylation status ≥ LOQ. LODs and LOQs of MS-HRM assays for *APC*, *BRCA1*, *CDKN2A*, *ESR1*, *HER2/neu* and *PTEN* are given in Table 2. The LOQ of the PSQ assay for *ESR2* is 5%. Due to the low LOD and LOQ of the assay for *PTEN* and the low frequency of methylation in tumors, tumor-adjacent and tumor distant-tissues were analyzed only randomly

Figure 3 shows the frequency of DNA methylation in the eight gene-specific regions investigated (seven promoter regions plus exon 2 of *CDKN2A*) in tumor, tumor-adjacent and tumor-distant tissues from breast cancer patients. Methylation levels obtained by MS-HRM analyses (*APC*, *BRCA1*, *CDKN2A*, *ESR1*, *HER2/neu* and *PTEN*) were divided into three subclasses (< LOD, < LOQ and ≥ LOQ), those obtained by PSQ (*ESR2*) into two subclasses (< LOQ and ≥ LOQ). Exon 2 of *CDKN2A* was the only region found to be methylated (methylation status ≥ LOD) in each (18/18) of the tumors. In contrast, the promoter of *CDKN2A* was only methylated in 28% (5/18). In addition to *CDKN2A* exon 2, the promoters of *APC* (83%, 15/18), *ESR1* (83%, 15/18) and *ESR2* (71%, 12/17) were frequently methylated in tumors. Figure 3 indicates that gene-specific methylation (methylation status ≥ LOD) was almost as frequent in tumor-adjacent and tumor-distant tissues as in tumors. However, exon 2 of *CDKN2A* more frequently showed a methylation status ≥ LOQ in tumor (94%, 17/18) than in tumor-adjacent (50%, 9/18) and tumor-distant (56%, 10/18) tissues.

**Fig. 4:**
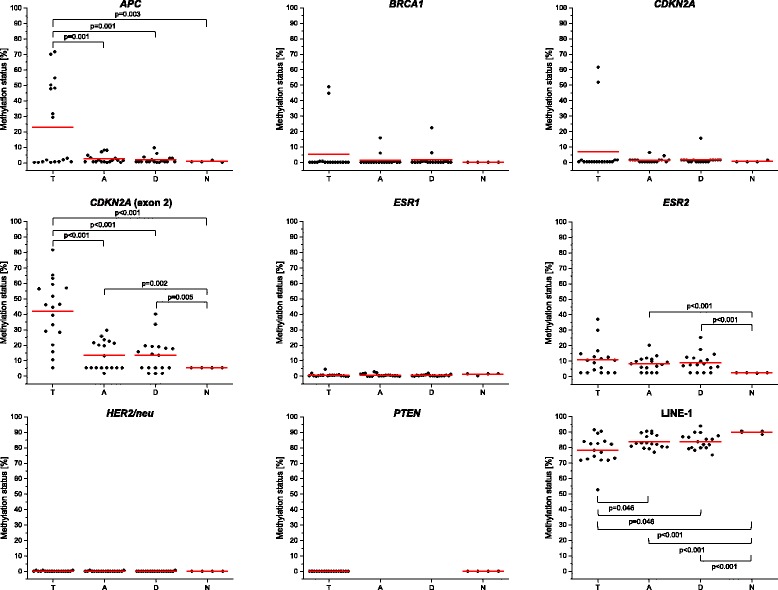
Dot density plots showing the distribution of gene-specific and global methylation levels. T: tumor tissue; A: tumor-adjacent tissue; D: tumor-distant tissue; N: normal breast tissue from healthy controls. *Red* line: arithmetic mean. Due to the low LOD and LOQ of the assay for *PTEN* and the low frequency of methylation in tumors, tumor-adjacent and tumor distant-tissues were analyzed only randomly

Figure 4 shows the distribution of gene-specific and global methylation levels in tumor, tumor-adjacent and tumor-distant tissues as well as in normal tissues from healthy women. With some exceptions, the promoter regions showed rather low methylation levels. The promoters of *ESR1*, *HER2/neu* and *PTEN* were almost unmethylated in each of the tissue samples investigated. Methylation levels ≥ 25% were only obtained for the promoters of *APC* in 44% (8/18), *BRCA1* and *CDKN2A* in 11% (2/18) and *ESR2* in 12% (2/17) of the tumor tissues and in addition for *ESR2* in 6% (1/18) of the tumor-distant tissues. In contrast, exon 2 of *CDKN2A* showed a methylation status *≥ *25% in 78% (14/18) of the tumors and 11% (2/18) of the tumor-adjacent and tumor-distant tissues.

The methylation status of *CDKN2A* exon 2 in tumors was significantly higher than in tumor-adjacent (*p* < 0.001) and tumor-distant tissues (*p* < 0.001) from the same patients and in normal breast tissues from healthy women (*p* < 0.001) (Fig. 4). In addition, the methylation status in tumor-adjacent (*p* = 0.002) and tumor-distant tissues (*p* = 0.005) was significantly higher than that in normal breast tissues from healthy women. Significant differences were also found between the methylation status of *APC* in tumors and that in tumor-adjacent tissues (*p* = 0.001), tumor-distant tissues (*p* = 0.001) and breast tissues from healthy women (*p* = 0.003) (Fig. 4). In case of *ESR2*, tumor-adjacent (*p* < 0.001) and tumor-distant tissues (*p* < 0.001) but not the tumors showed significantly higher methylation levels than normal breast tissues from healthy women. Tumors showed a significantly lower global methylation extent than tumor-adjacent (*p* = 0.046) and tumor-distant tissues (*p* = 0.046) and breast tissues from healthy women (*p* < 0.001). Significant differences were also found between the global methylation extent in tumor-adjacent (*p* < 0.001) and tumor-distant tissues (*p* < 0.001) and that in normal breast tissues.

We did not find a significant difference between the methylation status in tumor-adjacent and tumor-distant tissues from breast cancer patients, neither for the promoter regions, nor for exon 2 of *CDKN2A,* nor for the global methylation extent.

### Correlation between the methylation status in tumor, tumor-adjacent and tumor-distant tissues from breast cancer patients

For the following genes, a correlation was found between the methylation levels in tumors and those in tumor-adjacent tissues: *CDKN2A* (promoter) (*r* = 0.927, *p* < 0.001); *BRCA1* (*r* = 0.878, *p* < 0.001); *APC* (*r* = 0.706, *p* = 0.001), *ESR1* (*r* = 0.545, *p* = 0.019) and *ESR2* (*r* = 0.543, *p* = 0.024). In addition, we found a correlation between the methylation levels in tumors and tumor-distant tissues for *BRCA1* (*r* = 0.826, *p* < 0.001) and *ESR1* (*r* = 0.555, *p* = 0.017). In case of *BRCA1* (*r* = 0.994, *p* < 0.001), exon 2 of *CDKN2A* (*r* = 0.651, *p* = 0.003) and *ESR2* (*r* = 0.580, *p* = 0.015), methylation levels in tumor-adjacent tissues correlated with those in tumor-distant tissues.

For the global methylation status, correlations were found between tumor and tumor-adjacent tissues (*r* = 0.737, *p* = 0.001), tumor and tumor-distant tissues (*r* = 0.679, *p* = 0.003) as well as tumor-adjacent and tumor-distant tissues (*r* = 0.778, *p* < 0.001).

### Correlation between methylation levels of individual genes

Methylation levels of the *BRCA1* promoter strongly correlated with those of the *CDKN2A* promoter in tumors (*r* = 0.990, *p* < 0.001), tumor-adjacent tissues (*r* = 0.920, *p* < 0.001) and tumor-distant tissues (*r* = 0.963, *p* < 0.001). In tumors, the methylation levels of *ESR2* correlated with those of *APC* (*r* = 0.614, *p* = 0.009).

In addition, correlations were found with genes the methylation levels of which we had determined in previous studies [24, 31]. The methylation status of *ESR2* in tumors correlated with that of *ABCB1* (*r* = 0.614, *p* = 0.009) and *ABCG2* (*r* = 0.543, *p* = 0.030). The methylation status of *APC* in tumors was found to correlate with that of *MGMT* (*r* = 0.679, *p* = 0.003). In tumor adjacent tissues, the promoter methylation status of *ESR2* correlated with that of *MGMT* (*r* = 0.704, *p* = 0.002) and in tumor-distant tissues, the methylation status of *APC* correlated with that of *ABCB1* (*r* = 0.756, *p* < 0.001).

The methylation status of *CDKN2A* exon2 was, however, not found to correlate with any of the other regions investigated.

### Association between the DNA methylation status of individual genes with the age and clinicopathological parameters

For none of the genes investigated in the present study, we found a correlation between the age of the healthy women and the DNA methylation status in their breast tissues (based on breast tissues from four healthy women; in case of *CDKN2A* exon 2, breast tissues from seven women were taken into account). In tumor-distant tissues from breast cancer patients, the methylation status of *ESR1* was negatively correlated with the age of the patients at diagnosis (*r* = −0.673, *p* = 0.002).

Methylation levels of *CDKN2A* exon 2 in tumor tissues and also in tumor-distant tissues showed a weak but significant negative correlation with the proliferative activity of the tumor (*r* = −0.485, *p* = 0.041 and *r* = −0.498, *p* = 0.036, respectively). All tumors of the luminal A and luminal B subtype showed significantly higher (*p* < 0.001) methylation levels than the breast tissues from healthy women. Tumor of patient 12, belonging to the triple negative subtype, showed the lowest methylation status (methylation status < LOQ) of *CDKN2A* exon 2 among all tumors investigated. Since our set of breast tissue samples contained only one tumor of a triple negative subtype, we determined the methylation status of *CDKN2A* exon 2 in MDA-MB-231, a triple negative breast cancer cell line and compared it with the methylation status of the ER positive cell lines MCF-7 (luminal A) and ZR-75-1 (luminal B). In line with the tumor tissue from patient 12, the triple negative tumor cell line MDA-MB-231 showed a methylation status < LOQ, whereas the methylation status in MCF-7 and ZR-75-1 cells was 22 and 66%, respectively. A rather low methylation status was also found for the tumor from patient 18, belonging to the HER2/neu positive subtype.

### Gene-specific methylation patterns of the breast tissues analyzed

**Fig. 5:**
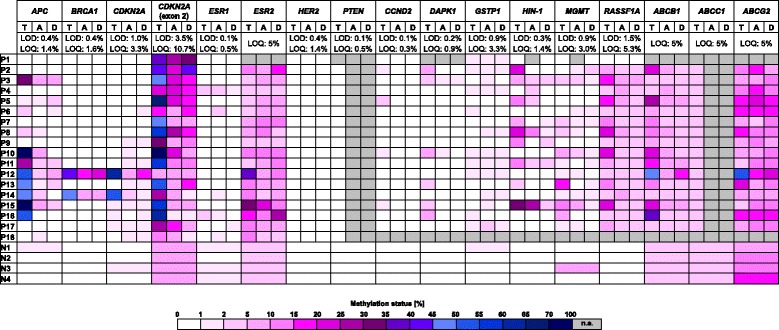
Gene-specific methylation patterns. P: patient; N: normal breast tissue from healthy controls; T: tumor tissue; A: tumor-adjacent tissue; D: tumor-distant tissue; LOD: limit of detection; LOQ: limit of quantification; n.a.: not analyzed. Data for *CCND2*, *DAPK1*, *GSTP1*, *HIN-1*, *MGMT* and *RASSF1A* [24] and *ABCB1*, *ABCC1* and *ABCG2* [31] has been published previously

**Fig. 6:**
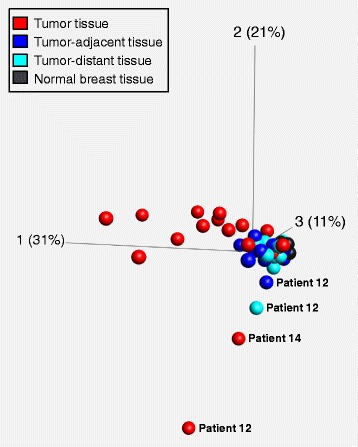
Principal component analysis (PCA) plot. The plot was generated by including methylation levels of 15 gene-specific regions in tumor, tumor-adjacent and tumor-distant tissues from 16 breast cancer patients and normal breast tissues from four healthy women. Due to incomplete data sets, promoters of *PTEN* and *ABCC1* and breast cancer patients 1 and 18 were excluded

Figure 5 shows the gene-specific methylation patterns of the individual tumor, tumor-adjacent and tumor-distant tissues from breast cancer patients and normal breast tissues from healthy women. In order to get a broader picture of the prevalence of pre-neoplastic DNA methylation changes in tumor-surrounding tissues in breast cancer patients, we included methylation levels that we had obtained for the same set of breast tissue samples in previous studies (*CCND2*, *DAPK1*, *GSTP1*, *HIN-1*, *MGMT* and *RASSF1A*: [24]; *ABCB1*, *ABCC1* and *ABCG2:* [31]). Gene-specific methylation patterns obtained for tumor tissues were obviously more heterogeneous with regard to both the frequency of methylation in the regions investigated and the extent of methylation than those obtained for tumor-adjacent and tumor-distant tissues from the same patients and normal breast tissues from healthy women. This observation was confirmed by principal component analysis (Fig. 6). Due to incomplete data sets, promoters of *PTEN* and *ABCC1* and breast cancer patients 1 and 18 were excluded in this evaluation. Figure 6 also demonstrates that the gene-specific methylation patterns of tumor, tumor-adjacent and tumor-distant tissues of patient 12 and tumor of patient 14 deviated from the patterns of the respective tissue specimens of other patients. As mentioned above, tumor of patient 12 belonged to the triple negative subtype, the tumor of patient 14 was of the luminal A subtype (ER status: moderately positive, PR and HER2/neu status: negative). However, expression of the estrogen receptor was only moderate (50%) and in consequence of chemotherapeutic treatment, expression of the estrogen receptor was found to be decreased to 10%. These facts indicate that the tumor of patient 14 was not a typical representative of the luminal A subtype.

**Fig. 7:**
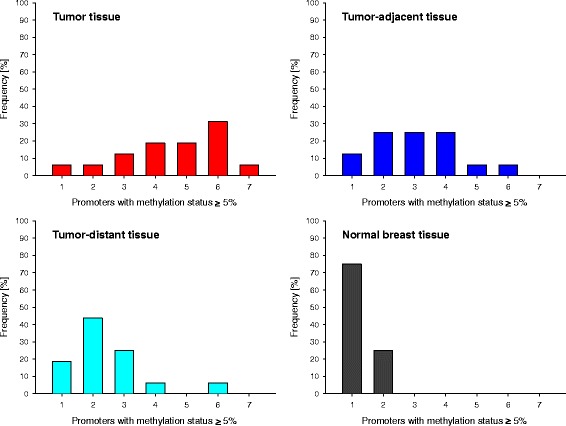
Frequency distribution of promoters with a methylation status ≥ 5% in patients and healthy controls. Frequency refers to tumor, tumor-adjacent and tumor-distant tissues from 16 breast cancer patients and normal breast tissues from four healthy controls. The promoters of the following genes were included: *APC*, *BRCA1*, *CDKN2A*, *ESR1*, *ESR2*, *HER2/neu*, *CCND2*, *DAPK1*, *GSTP1*, *HIN-1*, *MGMT*, *RASSF1A*, *ABCB1* and *ABCG2*. Due to incomplete data sets, promoters of *PTEN* and *ABCC1* and breast cancer patients 1 and 18 were excluded

Since hypermethylation of the promoter region of genes frequently leads to transcriptional silencing, for each of the breast tissue samples we calculated the number of promoters showing a methylation status ≥ 5%. Due to incomplete data sets, promoters of *PTEN* and *ABCC1* and breast cancer patients 1 and 18 were again excluded. Figure 7 shows the distribution of the frequency of promoters with a methylation status ≥ 5% in tumors, tumor-adjacent and tumor-distant tissues and normal breast tissues from healthy women. In tumor tissues, up to seven (out of 14) and in tumor-adjacent and tumor-distant tissues, up to six (out of 14) promoters showed a methylation status ≥ 5%, whereas in breast tissues from healthy women at most two promoters had a methylation status ≥ 5%. Figure 7 also indicates that in tumors most frequently six, in tumor-adjacent tissues two to four, in tumor-distant tissues most frequently two and in breast tissues from healthy women most frequently only one promoter had a methylation status ≥ 5%.

## Discussion

The present study provides methylation levels for the promoters of *APC*, *BRCA1*, *CDKN2A*, *ESR1*, *ESR2*, *HER2/neu* and *PTEN, CDKN2A* exon 2 and LINE-1 in tumor, tumor-adjacent and tumor-distant tissues from 18 breast cancer patients and normal breast tissues from four healthy women. In general, methylation levels were obtained by MS-HRM analysis. In case of *ESR2*, however, a MS-HRM method developed in-house suffered from low amplification efficiency. However, the pyrosequencing method developed alternatively was found to be applicable to determine the methylation status of *ESR2* in breast tissue samples. In contrast to pyrosequencing, which allows determining the methylation status of individual CpGs, MS-HRM assays give information about the average methylation status across all CpGs within the amplicon. However, from the shape of the melting curves, one can deduce if the region of interest is methylated homogenously or heterogeneously. In the present study, melting profiles indicating heterogeneous methylation were obtained e.g. for *CDKN2A* exon 2 in tumor tissues of patients 6, 14 and 18.

Among the eight gene-specific regions investigated (seven promoter regions plus exon 2 of *CDKN2A*), exon 2 of *CDKN2A* was the most frequently methylated one. In each of the tumors, it was methylated (methylation status ≥ LOD) and in 78% of the tumors, the methylation status was *> *25%. In breast tumors, methylation levels were significantly higher than in tumor-adjacent and tumor-distant tissues. With levels between the LOD (3.5%) and LOQ (10.7%) of the MS-HRM assay, breast tissues from healthy women were significantly lower methylated than the tissue specimens from breast cancer patients. From these results we can conclude that methylation changes in *CDKN2A* exon 2 are associated with breast carcinogenesis.

Moreover, we found some interesting associations between the methylation status in *CDKN2A* exon 2 and clinicopathological parameters. Weak but significant negative correlations were found between the methylation status in both, tumors and tumor-distant tissues, and the proliferative activity of the tumor. In addition, all tumors of the luminal A and luminal B subtype were found to show substantially higher methylation levels than the breast tissues from healthy women, whereas in two patients the tumors of which belonged to the triple negative and the HER2/neu positive subtype, respectively, the methylation levels were similar to those in healthy women. In order to underpin our finding on the triple negative tumor, we determined the methylation status of *CDKN2A* exon 2 in the triple negative breast cancer cell line MDA-MB-231 and compared it with that obtained for the luminal A cell line MCF-7 and the luminal B cell line ZR-75-1. In line with our results for tumor tissues, the methylation status of *CDKN2A* exon 2 in the cell line MDA-MB-231 did not differ from that in normal tissues from healthy women, whereas the cell lines MCF-7 and ZR-75-1 showed substantially higher methylation levels. However, since most of the tumors analyzed in the present study belonged either to the luminal A or the luminal B subtype, our results only can give a hint that hypermethylation of *CDKN2A* exon 2 is associated with the molecular subtype of the tumor.

To the best of our knowledge, the methylation status of *CDKN2A* exon 2 has not been linked to breast cancer so far. However, in a previous study, *CDKN2A* exon 2 has been found to be methylated in eight out of 16 oesophageal tumors, but in none of 16 normal tissue samples [30]. The methylation frequency was higher in late stage (III and IV) than in early stage tumors. In contrast to *CDKN2A* exon 1, which was found to be methylated only in five tumors, methylation of *CDKN2A* exon 2 did not correlate with expression of *CDKN2A* [30].

CDKN2A has a crucial function in cell cycle control and it is known to be inactivated in various types of cancer [41]. The prevalence of aberrations in promoter methylation and their role in inactivation of CDKN2A have been investigated for various cancer types [42, 43]. Findings for breast cancer [44–49] are, however, inconsistent. Some authors report higher frequency of *CDKN2A* promoter methylation in breast tumors [44, 45, 47–49], whereas others have not found a difference between malignant and non-malignant breast tissues [46]. Surprisingly, we found the promoter of *CDKN2A* less frequently methylated than exon 2. Only in two (patients 12 and 14) out of 18 tumors, the promoter of *CDKN2A* was higher methylated than in normal breast tissues from healthy women.

Moreover, tumors of patients 12 and 14 were the only ones among our sample set in which the *BRCA1* promoter was hypermethylated. BRCA1 is known to play an important role in the repair of DNA cross-links and double-strand breaks. Several papers have already described the association of *BRCA1* promoter hypermethylation with sporadic breast cancer [50–53]. Reported frequencies of *BRCA1* promoter methylation are quite different, ranging from about 9-15% [50, 51] to 59% [53]. In addition, reports on the association of *BRCA1* promoter methylation with the hormone receptor status of the tumor are inconsistent. In some studies, *BRCA1* promoter methylation has been linked to the ER and PR status of the tumor [48, 54], whereas others have not found a linkage between these two features [53]. In the study of Stefansson et al. [55] and in a recent meta-analysis [56], hypermethylation of the *BRCA1* promoter was significantly associated with triple negative breast cancer. By analyzing our set of tumor samples, we found an association between the *BRCA1* promoter methylation status and the hormone receptor status. As mentioned above, tumor of patient 12 belonged to the triple negative subtype, whereas tumor of patient 14 was of the luminal A subtype (ER status: moderately positive, PR and HER2/neu status: negative). However, since ER expression was only moderate and significantly decreased following chemotherapeutic treatment, tumor of patient 14 cannot be considered a typical representative of the luminal A subtype.

In the present study, *APC* was found to be frequently methylated in tumors, tumor-adjacent and tumor-distant tissues from breast cancer patients, but not in normal breast tissues from healthy women. Tumors showed significantly higher promoter methylation levels than tumor-adjacent and tumor-distant tissues. Our results refer to promoter 1A, which has been found to be methylated in various types of cancer [57], whereas promoter 1B, located about 30 kb upstream of promoter 1A [58], has been reported to be unmethylated [57]. In the present study, the methylation frequency of the *APC* promoter in tumors was 83%, which is higher than the frequencies reported in literature which were in the range from 36 to 55% [35, 59–63]. Most probably, this discrepancy is caused by the high sensitivity of our MS-HRM assay (LOD: 0.4%; LOQ: 1.4%). By determining the *APC* promoter methylation status in tumors and matched morphologically normal tissues from breast cancer patients, Van der Auwera et al. found significantly higher methylation levels in tumors which is in line with our results [63]. In contrast to other studies, in our set of samples, the *APC* methylation status was not associated with any of the clinicopathological parameters investigated. However, promoter methylation levels in tumors significantly correlated with those of *ESR2* and *MGMT*.

In contrast to previous papers reporting that promoter methylation of *PTEN* is a frequent event in breast tumors [64–67], in none of the tumors analyzed in the present study, the *PTEN* promoter was found to show a methylation status ≥ LOQ (although with 0.5%, the LOQ of the MS-HRM assay was very low). Results obtained by analyzing randomly selected tumor-adjacent and tumor-distant tissues of our sample set (data not shown) did not give a hint on *PTEN* promoter methylation in these tissue specimens.

Previous data on *ESR1* methylation in breast cancer is inconsistent. In some studies, the *ESR1* promoter was found to be frequently hypermethylated [54, 68–70] whereas according to others, methylation of the *ESR1* is a rather uncommon event in breast tumors [71]. In the tumor samples analyzed in the present study, the *ESR1* promoter was frequently (83%) found to be methylated (methylation status ≥ LOD). However, the MS-HRM assay applied was very sensitive (LOD: 0.1%, LOQ: 0.5%). In tumors, the methylation levels were almost as low as in the tumor-adjacent and tumor-distant tissues from the same breast cancer patients and normal tissues from healthy controls. In contrast to the study of Wei et al. [54], we did not find a correlation between the methylation levels of *ESR1* and *BRCA1*. However, in tumor-distant tissues, the promoter methylation status of *ESR1* was negatively correlated with the age of the patients at diagnosis.

We found the *ESR2* promoter frequently methylated in tumor, tumor-adjacent and tumor-distant tissues from breast cancer patients, whereas in normal breast tissues from healthy women, the methylation status was < LOQ (5%). However, methylation levels in tumor-adjacent and tumor-distant tissues, but not those in tumors were significantly higher than in normal breast tissues from healthy women. In tumor tissues, the promoter methylation levels of *ESR2* correlated with those of *APC*, *ABCB1* and *ABCG2*. In tumor-adjacent tissues, the methylation status correlated with that of *MGMT.* Rody et al. found the *ESR2* promoter methylated in two-thirds of invasive breast cancers [72]. Increased methylation levels were also detected in pre-malignant lesions. In another study, *ESR2* was methylated in 64% of breast tumors and 12% of adjacent normal tissues serving as control [28]. In contrast to our results, the methylation status of *ESR2* was correlated with that of *ESR1*.

We also determined the promoter methylation status of *HER2/neu*, although in literature there was no hint at the involvement of DNA methylation in gene regulation. Overexpression of HER2/neu is known to stimulate cell proliferation and tumor progression [29]. In none of the breast tissues analyzed, the methylation status was ≥ 1.4%, the LOQ of the MS-HRM assay applied. According to literature, overexpression of the oncogene *HER2/neu* is mainly regulated by gene amplification [29, 73].

In order to assess the global methylation extent, we determined the methylation status of the repetitive element LINE-1. Global DNA hypomethylation is known to be a common feature in cancer tissues including breast cancer [74]. Among our set of tissue samples, normal breast tissues showed a significantly higher global methylation extent than the breast tissue specimens from breast cancer patients. In addition, in tumor-adjacent and tumor-distant tissues the global methylation extent was higher than in tumors. A significant difference between tumor and tumor-adjacent breast tissues was also found by Cho et al. [75]. In previous papers, the methylation status of LINE-1 in tumors was associated with the age of the patients at diagnosis [76] or clinicopathological parameters, e.g. the ER status of the tumor [74]. In the sample set analyzed in the present study, the methylation status of LINE-1 was not associated with any of the clinicopathological parameters investigated.

Our results presented here and those published recently [24, 31] demonstrate that in breast cancer patients, tumor-adjacent and tumor-distant tissues frequently show pre-neoplastic gene-specific and global DNA methylation changes. Knowledge of the presence of a cancer field is of clinical relevance, because if the tissue showing pre-neoplastic lesions is not removed by surgery, it may develop into invasive carcinoma.

Results obtained by PCA demonstrate that gene-specific methylation patterns of breast tumors are more heterogeneous than those of tumor-adjacent and tumor-distant tissues from the same breast cancer patients and normal breast tissues from healthy women. Methylation patterns of tumor, tumor-adjacent and tumor-distant tissue of a patient with triple negative breast tumor were found to deviate from those of other patients. Our finding is in line with previous studies reporting distinct methylation patterns in triple negative breast tumors [77, 78].

Hypermethylation of the gene promoter is frequently linked to transcriptional gene silencing. Among our set of breast tissue samples, in tumor tissues up to seven (out of 14), and in tumor-adjacent and tumor-distant tissues up to six (out of 14) promoters showed a methylation status ≥ 5%, whereas in normal breast tissues from healthy women at most two promoters had a methylation status ≥ 5%. Previous studies have already reported simultaneous hypermethylation of a number of tumor suppressor genes in breast tumors [12, 35, 79–83], but also in matched normal tissues from breast cancer patients [12, 63].

## Conclusions

Our results demonstrate that in breast cancer patients, tumor-adjacent and tumor-distant tissues frequently show pre-neoplastic gene-specific and global DNA methylation changes. Among the eight gene-specific regions investigated, exon 2 of *CDKN2A* was most frequently methylated in tumors, tumor-adjacent and tumor-distant tissues from breast cancer patients. In tumors, *CDKN2A* exon 2 was significantly higher methylated than in tumor-adjacent and tumor-distant tissues. Normal breast tissues from healthy women showed significantly lower methylation levels than the tissue specimens from the breast cancer patients. From our data we can conclude that methylation changes in exon 2 of *CDKN2A* are associated with breast carcinogenesis. In a previous paper, hypermethylation of *CDKN2A* exon 2 has been associated with oesophageal cancer. To the best of our knowledge, aberrant methylation of *CDKN2A* exon 2 has not been associated with breast cancer so far. Further investigations will show if hypermethylation of *CDKN2A* exon 2 is limited to breast and oesophageal cancer, or also occurs in other types of cancer. Our results suggest that in breast cancer, the methylation status of *CDKN2A* exon 2 is weakly associated with the proliferative activity and the molecular subtype of the tumor. These findings have, however, to be confirmed in further studies.

## Abbreviations

ABCB1: ATP binding cassette subfamily B member 1
ABCC1: ATP binding cassette subfamily C member 1
ABCG2: ATP binding cassette subfamily G member 2
APC: Adenomatous polyposis coli
BRCA1: Breast cancer 1, early onset
CCND2: Cyclin D2
CDKN2A: Cyclin-dependent kinase inhibitor 2A
CpG: Cytosine-phosphatidyl-guanosine
DAPK1: Death-associated protein kinase 1
ER: Estrogen receptor
ESR1: Estrogen receptor 1, estrogen receptor α
ESR2: Estrogen receptor 2, estrogen receptor β
GSTP1: Glutathione S-transferase P1
HER2/neu: Human epidermal growth factor receptor 2
HIN-1: High in normal-1
IDC: Invasive ductal carcinoma
ILC: Invasive lobular carcinoma
LINE-1: Long interspersed element 1, retrotransposable element 1
LOD: Limit of detection
LOQ: Limit of quantification
MGMT: O6-methylguanine-DNA methyltransferase
MIB-1: Mindbomb E3 ubiquitin protein ligase 1
MS-HRM: Methylation-sensitive high resolution melting
PCR: Polymerase chain reaction
PR: Progesterone receptor
PSQ: Pyrosequencing
PTEN: Phosphatase and tensin homolog
RASSF1A: Ras association domain family member 1
S/N: Signal-to-noise ratio
T_a_: Annealing temperature
